# A289 MAJOR ADVERSE CARDIAC EVENTS (MACE) IN PATIENTS WITH A HISTORY OF LIVER TRANSPLANTATION: A NATIONWIDE ANALYSIS USING THE NATIONAL INPATIENT SAMPLE

**DOI:** 10.1093/jcag/gwad061.289

**Published:** 2024-02-14

**Authors:** M Hussain, A Teriaky, D Hudson

**Affiliations:** Western University, London, ON, Canada; Western University, London, ON, Canada; Western University, London, ON, Canada

## Abstract

**Background:**

Liver transplantation is the preferred treatment for end-stage liver disease. However, in around one-third of transplant recipients cardiovascular disease remains to be a significant contributor to morbidity and mortality.

**Aims:**

Using data from the national inpatient sample (NIS), this study aimed to assess the prevalence and patient characteristics of MACE in those who underwent liver transplantation.

**Methods:**

The study used a cross-sectional design to analyze NIS data, comparing MACE prevalence in hospitalized liver transplant patients. MACE included heart-related events. Patients with MACE and liver transplant history were identified using 2013 NIS data and ICD-9-CM codes. Baseline characteristics were compared, and association strength was assessed via logistic regression.

**Results:**

Out of 33,725 post-liver transplant patients, 26.1% (8,805) had prior MACE. Those with MACE had higher rates of comorbidities: type 2 diabetes (odds ratio (OR) 1.90, 95% CI 1.69-2.13, p ampersand:003C 0.001), hypertension (OR=2.07, 95% CI 1.83-2.34, pampersand:003C 0.001), dyslipidemia (OR=2.27, 95% CI 1.97-2.60, pampersand:003C 0.001), and morbid obesity (OR=1.69, 95% CI 1.39-2.04, pampersand:003C 0.001). Smoking wasn't significantly linked (p = 0.738). Hospitalization with MACE increased in-hospital mortality (3.25%, p ampersand:003C 0.001).

**Conclusions:**

These findings highlight the importance of proactive cardiovascular risk factor management to minimize the morbidity and mortality of MACE in this patient population.

1: Baseline characteristics of patients admitted to hospital with or without MACE after liver transplantation.

**Funding Agencies:**

None

**Table:** 

Characteristics	MACE (n = 8,805)	No MACE (n = 24,920	P-value
Age (years), mean (SE)	62.92 [0.360]	50.92 [0.6493]	ampersand:003C0.001
Gender, n (%)			0.002
Male	5,538 (62.9	14,553 (58.4)	
Race, n (%)			ampersand:003C0.001
White	6,621 (75.2)	16,704 (67.0)	
Black	630 (7.15)	2,684(10.77)	
Hispanic	955 (10.85)	3,848 (15.44)	
Asian/Pacific Island	273 (3.1)	740 (2.97)	
Native American	32 (0.36)	82 (0.33)	
Other	294 (3.34)	862 (3.46)	
Location, n (%)			ampersand:003C0.001
Rural	528 (6.0)	1,301 (5.22)	
Urban (non-teaching)	2,346 (26.64)	5,136 (20.61)	
Urban (teaching)	5,931(67.36)	18,483 (74.17)	
Co-morbidities			
Diabetes	4,450 (50.5)	9,500 (38.1)	ampersand:003C0.001
Hypertension	6,300 (71.6)	15,010 (60.2)	ampersand:003C0.001
Lipid	2420 (27.4)	3895 (15.6)	ampersand:003C0.001
Smoking	730 (8.29)	2335 (9.37)	0.7384
Obesity	945 (10.7)	1810 (7.26)	ampersand:003C0.001
Length of Stay, days			ampersand:003C0.001
Mean, SE	7.36 [0.382]	4.98 [0.116]	
			
Mortality, n (%)	286 (3.25)	194 (0.78)	ampersand:003C0.001

